# Measurement of Progress in the Environmental Area: Poland against the Countries of the European Union

**DOI:** 10.3390/ijerph20010563

**Published:** 2022-12-29

**Authors:** Ewa Mazur-Wierzbicka

**Affiliations:** Institute of Management, University of Szczecin, 70-453 Szczecin, Poland; ewa.mazur-wierzbicka@usz.edu.pl

**Keywords:** environmental area, indicators, environmental index, Poland, European Union

## Abstract

The initiatives taken by the European Union (EU) and the strategies it adopts aim to achieve sustainable development in a long-term perspective. This, however, requires continuous and consistent efforts to minimise the pressure on the natural environment. By obliging and encouraging Member States to take action in the environmental area, the EU wants to be a leader in conducting the green energy transformation. The main aim of the article is to assess the level of advancement of the EU countries (taking into account their division into two groups: EU-13 and EU-15) in making efforts to preserve the natural environment, with a particular emphasis on the position of Poland among the EU countries. An environmental index (EI) was used to make comparisons between EU countries. This index was designed on the basis of selected indicators during a statistical analysis. The Principal Component Analysis and the cluster analysis are employed in the article. This analysis puts forward a claim that it was mainly the countries of the EU-15 (Denmark and Sweden, in particular) that ranked highest in the environmental area in the period analysed and thus are the most advanced in terms of taking action for environmental protection—they took leaders’ positions. Romania and Bulgaria took the lowest positions in these comparisons. Poland’s score was low for the EI in the period analysed compared to the EU-28 countries. Establishing a more effective environmental policy in Member States with the lowest results is most crucial. The consistency of ordering countries according to the EI was noticeable in the period investigated. This proves the stability of the positions occupied by the EU-13 and the EU-15 group. Even though the European Union has made great progress with regard to the protection of the natural environment and green transformation, there is still much more to be done to increase the efficiency of resource use, waste recycling, energy efficiency or RES sharing in energy mixes.

## 1. Introduction

In the era of irreversible climate change [1], caused by, for example, the overpopulation and increased economic growth of new industrialized countries (South Korea, India, Malaysia, Philippines, Singapore, etc.), environmental pollution and the depletion of natural resources, the question of environmental protection (which has been addressed more and more frequently since the 1960s) is being more and more emphasized. These factors pose a challenge. This applies to the macro-, meso- and micro-scale. It is manifested, e.g., in the creation and development of concepts in which aspects that relate to the natural environment take an essential place. The most important ones include the concept of sustainable development (SD) [2,3,4] and the concept of a circular economy (CE) [5,6,7].

Initiatives taken up on the international arena, especially those after 2000, show that we must define the economy anew and build a simultaneous economic, social and environmental (ecological) balance under the newly advancing model of socio-economic progress called the “green development of the economy.” This progress places a particularly strong emphasis on environmental issues [8,9]. Within the European Union (EU), it is reflected in the EU’s 7th Environment Action Programme, whose key aims include the “protection, conservation and enhancement of the EU’s natural capital” and “turning the Union into a resource-efficient, green, and competitive low-carbon economy” ([10], pp. 182–186). It is then continued in the EU’s 8th Environment Action Programme (with a 2030 perspective) under six priority areas [11]. Additionally, the EU has introduced other documents that support the green development of the economy in the EU. For example, the European Commission adopted a new Circular Economy Action Plan in March 2020 that defined initiatives for the life cycle of products, it adopted the Zero Pollution Action Plan in May 2021 and it launched the Fit for 55 Package in mid-July 2021.

The new model should rely on the experience of environmental economics and ensure appropriate relations between the economy and ecosystems. It is supposed to be the opposite of the model of the economy based mainly on the use of fossil fuels and other non-renewable resources. The emerging theme of “greening the economy” is examined on many platforms [12]. It encompasses many issues, e.g., the development of pure technologies [13,14], renewable energy sources [15,16,17], the improvement of energy and material efficiency [18], changing the model of consumption and transforming production into a more sustainable one [19], an integrated product policy [20], Green Public Procurement [21,22], the development of new environmentally friendly industries, green jobs [23], or a green tax reform [24,25,26]. Therefore, innovation with a focus on the processes of managing green knowledge and its impact on green investment and green technologies plays an important role here [27,28,29]. This translates not only into the environmental area of sustainable development, but also into its socio-economical dimension [30] and the economies’ striving towards CE [31].

Given the above, it is paramount that the focus be given to the environmental issues that fit within the created environmental area (dimension)—assuming that it is one of the fundamental areas of SD (apart from the social or economic realms). This plays an important role, not only in the context of ecological policies or health protection, but also in the socio-economic progress.

Neglecting or ceasing environmental actions often results in non-reversible changes in the natural environment (e.g., air, water or soil pollution, depletion of resources, or climate change) and serious consequences in the economic and social dimensions (quality of life, welfare of the society).

In order to make rational decisions that adequately address the problem, the actions under the environmental area must be focused on collecting adequate information, both qualitative and quantitative, on diagnosing the current situation, and on making comparisons between the progress in each project undertaken [32]. Two indicators are used for this purpose. They let us better understand the problems that appear in the environmental dimension and the changes that occur in it from the perspective of the implementation of sustainable development. As Meadows claims ([32], p. 2), “indicators arise from values (we measure what we care for) and create values (we care for what we measure).” They are used extensively by countries, organizations and decision-makers due to their capability to sum up and condense a great deal of information about the complexity of our dynamic environment and present it in the most comprehensible way [33,34]. It is also commonly agreed that “the main quality of an indicator is its capacity to take into account the most precise manner possible, a phenomenon which generally tends to be complex in nature” ([35], p. 13). Employing quantitative indicators allows us to quantify facts and to generalise results for a specific group. A well-built indicator transforms basic statistical information to better understand the problem or dimension and to get a readable image of the entire system, including its interrelations and compromises [36]. It often reflects critical problems or challenges that await us.

KEI ([37], p. 1) claims: “Indicators and composite indicators are increasingly recognized as a useful tool for policy making and public communication in conveying information on countries’ performance in fields such as environment, economy, society, or technological development”.

Because tracking and interpreting changes in a large number of indicators and making comparisons between the values of many indicators that compare countries may be problematic, researchers design complex indicators/indices (composites). These are single, easily interpretable values that add multidimensionality to the examined problem. They reduce the pool of information that was provided by numerous dashboard indicators to the level that makes the analysis comfortable and ensures a uniform scale, on the basis of which we may measure countries’ comparative results [38].

As is the case with other tools, composite indices also bring difficulties and a likelihood of error. These may include errors that relate to weight or compensability, errors in synthesis and comparability, conceptual errors, or difficulties with specifying an appropriate method of aggregation or of the purpose of combining many predictive variables into a composite index. These limitations have been noticed and discussed [39,40,41,42]. With these in mind, we need to employ all research diligence and reliability in creating an index by relying on statistical reliability, the objectivity of criteria for selecting an appropriate method of aggregation that allows comparability [43,44], simplicity and transparency.

When reviewing the literature in terms of measuring environmental aspects, we can see that an intensification of the work on designing adequate indicators has been ongoing since the 1970s. Many studies have emerged so far that address specific environmental subjects or the entire economy-energy-environment system, or that provide an analysis that covers individual countries/regions/branches of many counties/regions. The creation of frameworks and theories for computing the environmental index was already addressed by Ott [45]. Other researchers also calculated the environmental index (EI) [46,47,48,49,50] and worked on creating new frameworks so that the EI may present condensed environmental information [51,52,53,54].

Some of the designed environmental indices are international, such as the Environment Quality Index [55], the Index of Environmental Friendliness [53], the Ecological Footprint [56], the Living Planet Index [57], the Eco-efficiency Indices [58,59], the Environmental Pressure Indicators [60], the Environmental Adjusted Domestic Product [61], the Environmental Sustainability Index [62,63,64], the Environmental Vulnerability Index [65] or the Environmental Performance Index [63,66,67,68]. Others focus on individual countries or regions, taking into account their specific characteristics, such as the environmental index for Great Britain [48] or the Environmental Policy Performance Indicator for the Netherlands **[41]**.

It needs to be noted that some indicators are being approached in a few different ways. It may be seen in the case of the Ecological Footprint indicator, around which many different concepts of indicators have been created—for example, the Material-Input-Per-Service [69], Ecoindex™ [70] and Sustainable Process Index [71,72].

Some research also focuses on indicators in which the environmental element is one of the components. From the perspective of the content of this paper, this applies to the indicators used to measure sustainable development, under which the environmental aspect is highlighted for example a barometer of sustainability [73]. It comprises two parts: ecosystem well-being, in the environmental aspect, and human well-being, in terms of social aspects. This clearly shows that improvement in both dimensions is equally important to achieve sustainable development. 

On the other hand, when we refer to measuring sustainable development, we need to point to sustainable development goals (SDGs) and their relevant indicators (here: those that refer to the environmental area). Given the spatial area that this article covers, the indicators associated with the implementation of the EU policy have been identified (Table 1).

The European Union is where the subject matter of environmental protection has long been a priority. It may be seen in the development of European environmental law (also in relevant documents), in the evolution of EU’s green policy (the first such programme has been effective since 1973, and since 2 May 2022, the eighth programme for environmental action has been in force as a legally justified common EU programme for the policy of environmental protection till 2030), or in the system of financing it. It is quite important that the EU is leading the implementation of the energy transformation process, which, at the moment, constitutes a fundamental element that makes up the broadly understood environmental policy [74,75,76].

Environment-related international obligations are today essential elements of creating the economic and social reality. It should be especially important for countries that make up the EU, for those which are its founding members and for those who joined it just a few years ago. For some countries, accession to the EU was one of the more essential events which complemented the transformation process initiated in the 1980s and 1990s. Poland is an example of such a country. Catching up with different issues, including those in the environmental aspect, was a great challenge, despite the implementation of further ecological policies and meeting the obligations under the “Environment” area laid down in the Accession Treaty, as well as other obligations resulting from documents adopted by the European Commission. EU membership was an opportunity for Poland to take the necessary steps to rationally manage its natural resources, counteract and prevent harmful effects to the natural environment, and restore the natural environment or its elements to a proper condition or climate protection.

It is crucial to see whether Poland has used the opportunity it was given. In the context of this article, this opportunity will refer to the environmental protection aspects. It is also interesting to assess the degree of Poland’s advancement in the environmental area against other EU countries, including those that became EU Members in 2004. Carrying out such an assessment will allow for the specification of the pace at which Poland is taking environmental steps, compared to the other EU countries, and whether they are sufficient for today. The focus here is on Poland because it is the largest of the “new accessions” in terms of area and population alike. On top of that, being accepted as part of the EU meant that Poland had to meet many of its requirements relating to environmental protection. The negotiations around the “Environment” area were one of the most difficult; Poland was given a total of 10 transitional periods, which was the most among all the other countries that were applying for EU citizenship at the same time [77]. Poland is also the first state out of the countries with a centrally-planned economy (Central and Eastern Europe) to transition to a free-market economy. It is also important that documents that are fundamental to Poland refer to sustainable development (Article 5 of the Constitution of the Republic of Poland; Article 13 of the Environmental Law “The national environmental policy shall mean a set of actions to create the conditions necessary for the implementation of environmental protection, in accordance with the sustainable development principle”). The fact that the Polish energy policy is largely based on conventional energy sources is also crucial. Poland is the largest hard coal and second largest lignite producer in the EU [78].

This paper focuses on the macro level. In total, 28 Member States of the European Union (EU-28) are adopted as the research matter. Additionally, two groups are also identified: the EU-13 countries (those who had become EU members before 2014) and the EU-15, the countries of the “old Union” (together with Great Britain due to the time of the analysis). Given the above division, a particular focus is given to one country, that is, Poland, which has been a member of the EU since 2004.

The adopted research period is 2010–2020—that is the time when the Europe 2020 strategy was effective. This strategy strongly emphasised issues relating to environmental protection, assuming, under the “sustainable development” priority, support for an effective economy that uses limited resources rationally and that is environmentally friendly. Such an assumption was allocated adequate priority goals, that is, reduction by 2020 of greenhouse gas emissions by 20% compared to those of 1990, increasing the share of renewable energy sources in our final energy consumption to 20% by 2020, with a 20% increase in energy efficiency by 2020 [79]. The European Commission’s document, Europe 2020, also lists flagship initiatives that correspond with these priorities. From the environmental perspective, the Resource Efficient Europe initiative was especially important. It focused on actions to decouple economic growth from resource use and on a transformation towards a low-emissions economy that uses the potential of renewable energy sources more. We must also note that the assessment of the degree of the implementation of the Europe 2020 strategy (naturally, in consideration of environmental factors) is addressed in numerous studies. Such an assessment is made, for example, on the basis of aggregated measures while applying a different methodological approach, that is, it shows an aggregated level of achievement by individual countries of all measures or environmental indicators [80,81,82,83,84,85,86]. It compares the values of national individual monitoring indicators with their target values—goals set at the EU level until 2020 [87]. It also juxtaposes the values of individual national monitoring indicators with their target values for a given country for 2020 [87].

Commentators claim that a significant strengthening of the environmental dimension took place as part of the 2020 strategy, if only by the emergence of the Green New Deal direction. While it has been in force, a number of projects and documents dedicated to the protection of the natural environment have been taken up, and they include: the Sixth Environment Action Programme [88], the Thematic Strategy on Sustainable Use of Natural Resources [89], Closing the loop—An EU action plan for the Circular Economy [90] and A new Circular Economy Action Plan [91]. In some of them, the European Commission imposed an obligation on Member States to carry out processes that involve minimising the negative impact on the natural environment. They also point to the aspect of monitoring many angles directed on environmental issues (relevant indicators in the Europe 2020 strategy, CE monitoring framework, etc.).

Each EU country is facing many challenges relating to sustainable development, which is strongly related to the CE they are implementing and to joining in the green transformation. The analysis of the relevant literature proves that the environmental impact is still the greatest challenge. It impacts the EU countries’ ability to achieve their targets of sustainable environmental development by meeting SD goals. It is one of the reasons for the recent increased demand for integrated environmental information. It is used to assess the results of sustainable environmental development in individual countries.

The effects of changes in the natural environment are more and more tangible. It seems that, in a world that is becoming more and more globalised, each tool that serves to assess the changes occurring in this area in individual countries or groups of countries (bearing in mind their specific characteristics) becomes useful. Even though there is research on measuring SD or on environmental performance (quoted above), there are still many possibilities in this field because the context of the dimensions of sustainable development, and the environmental dimension for countries or any given organization, is complex and diversified [92]. The literature review shows that, so far, no assessment of the EU countries broken down into the EU-13 and the EU-15 groups has been made in the context of assessing the environmental area. In addition, no analysis has been carried out so far from the perspective of Poland, the largest EU Member among the “new EU accessions,” since 2004. An analysis that classifies countries into the “new Union” and the “old Union” seems reasonable, even from the perspective of the differences in the economic development that occur between them. The usefulness of the analysis in the context of implementing new financial perspectives is also not quite irrelevant given the above. Choosing for this study EU countries (and the division employed) which so far have not been subject to such an investigation in the environmental area warrants this paper’s originality.

Because qualitative indicators facilitate the quantification of facts and the generalisation of results for a specific group and due to the significance of this subject matter, an indicator is designed in this paper that is easy to use and gives easily-interpretable results. It also gives an opportunity to identify key aspects of the massive quantity of information and helps decision-makers notice the emerging models and regularities. The construction of the index focused on the specific characteristics of the EU from the perspective of the environmental area, which is one of three fundamental dimensions of sustainable development. This was motivated by a lack of a single indicator that would reflect the state of affairs in the approach to environmental issues from the perspective of the EU countries. The EI is the second factor that proves the originality of this paper. 

Therefore, from the perspective of the green transformation that is happening now in the European Union, which is crucial for ensuring the EU’s long-term stability, it is important that research is carried out into how advanced individual EU countries are in environmental issues. It is important in this context to understand the progress that Member States have made in the environmental dimension. Designing a composite index based on adequate indicators can create a tool that allows for the monitoring of countries’ progress in the fight against negative changes in the natural environment. This tool also facilitates benchmarking in EU Member States. The EI designed here is supposed to be one of the tools that flags environmental conditions and trends and may be useful in helping political decision-makers make environmental decisions and have well-informed opinions.

The awareness of belonging to individual groups of countries, or the position in the ranking, shows decision-makers—politicians—the direction in which their country is going in the environmental area. This makes it possible for them to take an interest in the environmental policies of leading countries using their good practices and to compare environmental policies within individual groups. It allows for the identification of major environmental problems in selected groups of countries and joint efforts to remove them and to strive to create and implement effective and efficient solutions and actions for problematic hotspots in the environmental area. Therefore, one of the fundamental goals of this tool is to make a contribution to the implementation of improvements and to model the design of environmental policies by monitoring them, making consistent assessments and supporting the green transition of EU countries. The identification of groups of similar countries also provides an opportunity to direct EU investment on specific projects pursued by these countries in the investigated area.

Given the above, it seems important to make comparisons between Member States for the implementation of actions for the natural environment, identifying the leaders and EU countries which have the most to catch up on in the environmental area.

This paper focuses mainly on the assessment of the level of advancement of the EU countries in terms of actions for the natural environment (in the environmental area), including an analysis that compares Poland with the EU countries (taking into account their division into groups of EU-13 and EU-15 countries). It is the main aim of the paper. In order to do so, an adequate environmental index was designed. The following two research tasks corresponding to the main goal were also presented in the study:

Task 1—The assessment of the degree of Poland’s advancement in the implementation of actions in the environmental area against EU countries (taking into account the division of the EU countries into groups of EU-13 and EU-15 countries) in 2010–2020. This research task was executed in the following stages:

- The identification and allocation of indicators of the environmental dimension;
- The construction of the environmental index;
- The assessment of the development of the environmental index in Poland against the EU countries;
- Ordering and grouping countries with similar levels of the environmental index.

Task 2—The classification of the EU countries on the basis of the environmental index using the methods of the factor analysis (Principal Component Analysis) and cluster analysis. This task was executed in the following stages:

- Component analysis in blocks of variables for the environmental index;
- Comparative analysis of the values of components in selected groups of EU countries;
- Grouping EU countries into relatively uniform classes in terms of their similarities, that is, establishing the place of the EU countries in two clusters.

This analysis (classified according to the EI, grouping and factor and cluster analysis) was used to put forward a claim that it was mainly the countries of the EU-15 (Denmark and Sweden, in particular) that ranked highest in the environmental area in the period analysed. Thus, they are the most advanced in terms of taking action for environmental protection—they took the positions of leaders. Greece scored the lowest among the EU-15 countries, with EI values that were lower than many of the EU-13 group. Poland scored relatively low for EI compared to the EU-28 countries and thus was placed in an unsatisfactory position in the general classification, belonging to an identified cluster (II) with countries that are not leading in their environmental actions. This is for two reasons: gross negligence in the environmental protection field before the transformation period and the Polish economy (especially energy)’s reliance on hard and lignite coal.

## 2. Materials and Methods

An in-depth literature study and statistical analysis based on processed secondary sources are the basis for the inference here and for the achievement of research tasks. Deduction and induction were used in the research process and comparative analysis in time and space was carried out. When selecting research methods, the author was mostly guided by the possibility of having a broad look at the adopted research subject matter.

Following a thorough review of literature and a review of available data, a decision was made to use “environment-related” indicators in the analysis that were proposed by the European Commission to monitor CE and SD progress, Eurostat and OECD databases (including those that concern monitoring advancement in the implementation of the strategy of green growth or green transformation). While the literature does supply many criteria for selecting indicators, there is no single standard approach to choosing them [93].

The starting point was to verify selected collections of indicators and to eliminate those that carry the same information load or have the least diagnostic value. Assuming that one of the most essential elements of taxonomic procedures involves a selection of diagnostic variables used first to build a synthetic measure when selecting indicators, the following criteria were taken into consideration: availability, ease of interpretation, universality, changeability, significance and degree of correlation [94,95].

These are criteria that are applied to assess adequacy of indicators and to select indicators, and they are constructed on the basis of the Bellagio STAMP principles and OECD criteria [96,97].

A set of 37 potential diagnostic variables was selected for working on the EI out of the total of 57 indicators examined. They were presented to 7 experts that deal with the issues of the natural environment and sustainable development [63,98].

Experts were asked to assess the significance and usefulness of the indicators (Table 2) selected for the research together with the accompanying arguments in order to choose the most representative primary variables. As a starting point, they assumed associating the indicators with EU’s policy for the environmental area of DS, with aspects of which the EU places the main emphasis on in the green realm.

“Three” was considered the minimum average result of the indicator’s significance, thus its usefulness for the analysis. All indicators at this stage that fail to score the minimum of three from all experts were eliminated from further analysis. 25 indicators qualified for the second stage because some experts could not agree on the score. Another round was run for them, along with adequate questionnaires to eliminate 5 indicators. In this round, the procedure for the assessment was kept the same as in stage 1. After analysing all assessments and reasoning, 20 potential diagnostic variables were selected, which were a starting point for further analysis.

Ultimately, a set of 20 potential diagnostic variables was singled out for further selection:

1. Generation of municipal waste per capita
2. Recycling rate of municipal waste
3. Composted municipal waste
4. Sulphur oxide emissions (SO_x_)
5. Nitrogen oxide emissions (NO_x_)
6. Emissions of non-methane volatile organic compounds
7. Ammonia emissions (NH_3_)
8. Percentage of population who live in household that experience noise-related inconveniences
9. Primary energy consumption
10. Share of renewable energy in gross final energy consumption
11. National gross energy consumption
12. Resource productivity
13. Energy productivity
14. Energy dependence
15. Raw material consumption
16. Circular material use rate
17. Final energy consumption
18. Resource productivity and domestic material consumption
19. Greenhouse gas emissions per unit of consumed energy
20. Greenhouse gas emissions per capita

A set of data selected like this guarantees considerable capacity for diagnostics, as it is not too overwhelming and, at the same time, it ensures a relatively universal description of the object investigated.

Each of the indicators was assigned the same weight—this resulted, for example, from information obtained during expert interviews, from arguments presented when selecting the indicators and from the analysis of examples of building indices that feature in relevant literature [63,99]. The investigation also takes into account the fact that experts’ assigning weight to indicators, also environmental ones, may cover subjective factors, which may be determined by, for example, their various specializations. This, in turn, may affect reliability and objectivity of the analysis. Additionally, when there is no fully consistent information from experts or if there is no mechanism to specify relative significance for various environmental variables, it is acceptable to assume equal weights [48,100]. 

Variable 14 was a negative destimulant. This is why, following the procedure adopted in this study, it became necessary to transform it (to unify the set of potential variables) according to the following formula:

x’_ij_ = − x_ij_ + max(x_ij_) (1)

where: x_ij_—variable values for objects.

Thus, this variable became a stimulant, and this is how it was examined in further calculations.

Taking into account the criterion of sufficient spatial variability when selecting variables, the author was guided by a classic variation coefficient and treated its location-related equivalent as an additional measure (the positional coefficient of variation applied to variation in half of the population studied—individuals with values of a given feature below the first and above the fourth quartile were rejected). V_j_ > 10% was adopted as the critical value of the first of the coefficients mentioned. If the positional coefficient pointed to a rejection and the classic one did not, then the variable was not rejected.

However, when making such decisions, the procedure proposed by K. Kukuła [101] was followed. He proposes that one more coefficient be used in the decision-making process next to the coefficient of variation. He calls it a coefficient of a relative amplitude of variations of variable W_j_:

(2) $$\begin{array}{c} A(w_{j})\;=\frac{{\max\;w}_{\mathrm{ij}}}{{\min\;w}_{\mathrm{ij}}}\\ \;\;\;\;\;\;\;\;\;\;\;\;\;\;\;\;\;\;\;\;\;\;\;\;\;\;\;\;\;\;\;\;\;{\min w}_{\mathrm{ij}\;\neq \;0} \end{array}$$

where: A(W_j_)—coefficient of a relative amplitude of variable W_j_ fluctuation.

It is believed that only a joint meeting of the following two requirements:

- 0 ≤ V(W_j_) ≤ 0.1
- 1 ≤ A(W_j_) ≤ c,

where: V(W_j_)—a classic coefficient of variation for indicator W_j_,
c—constant (assumed by default),
may evidence that a given variable should not become part of a collection of variables that serve further analysis.

Given the above, classic and positional coefficients of variations were calculated for the potential variables examined. Considering that the assumed critical value of the coefficient of variation was not exceeded in the analysis, variable 19 was rejected. At this stage, a second rejection criterion for components of the fluctuation amplitude was also taken into account in parallel. The need to eliminate variable 19 was confirmed.

The reduced set of variables was, in turn, analysed for validity. To do so, a classic coefficient of asymmetry was calculated for individual variables according to the following formula:

(3) $${\tilde{A}}_{s}=\frac{n^{2}}{\left(n-1)(n-2\right)}\frac{M_{3}}{s^{˜3}}$$

where:

(4) $$M_{3}=\frac{1}{n}\overset{n}{\underset{i=1}{\sum }}{\left(x_{i}-\bar{x}\right)}^{3}$$

where:

${\tilde{A}}_{s}=$—classic coefficient of asymmetry,

*n*—number of countries analysed,

$\bar{x}$—arithmetic mean,

*M*_3_—third central moment.

The great majority of variables displayed an undesirable type of asymmetry (for stimulants, it was a left-skewed asymmetry, and for destimulants, a right-skewed rather than left-skewed asymmetry). Only 6 of the variables: 1, 2, 3, 10, 14 and 20 met this criterion. An additional analysis was carried out because of this. It used a positional coefficient of asymmetry calculated according to the formula:

(5) $$A_{q}=\frac{\left(Q_{3}-Me\right)-\left(Me-Q_{1}\right)}{Q}$$

where:

*Q* = *Q*_3_ *− Q*_1,_
*Me*—median,
*A_q_*—positional version of the coefficient of asymmetry.

It is a supplementary measure; it specifies the direction and force of the asymmetry of individual units that are in the second and third quartile of the area of variability, thus in a narrowed area.

In this case, too, the bulk of the variables qualified for rejection. However, by taking into account an additional criterion, we could expand the pool of variables at this stage of selection (next to those qualified above) with another 3: 6, 12 and 13. Therefore, 9 potential variables qualified for the next selection stage.

The set of variables that had not been reduced until then was analysed, in turn, for their degree of correlation. Variable clusters were identified (the fact of exceeding the critical value of coefficients of correlation r* = 05* was taken into account in the procedure of their identification). Central and satellite features were identified within the clusters. Isolated features were also identified.

Because different sets of central and isolated attributes emerged in different periods, the frequency of emergence of a given central or isolated variable in the entire research period was taken as a basis for deciding ultimately which variables to choose for the final set. The following variables were rejected at this stage: 3, 6, 12 and 13.

In the end, 5 indicators qualified for the set of diagnostic variables to build the EI (Table 3). The majority of variables are distimulants; indicators 2 and 10 are exceptions (stimulants). Variable 14 was transformed into a stimulant already at the outset, thus was treated as such. To streamline the set of variables, distimulants were transformed into stimulants, following the formula:

x_ij_ = 1/x’_ij_ (6)

i = 1, 2, …n
j = 1, 2, …p,
where:
n—number of samples,
p—number of variables.

No nominants were identified among the variables.

The groups of diagnostic variables identified were used to set the synthetic measure of the EI for each of the countries (and groups of countries—an index median in a given group). The next step was to divide the set of objects investigated according to formulas:

- Group I (very good): $z_{i}\geq \bar{z}+s_{z},$
- Group II (good): $\bar{z}+s_{z}>z_{i}\geq z,$
- Group III (poor): $\bar{z}>z_{i}\geq \bar{z}-s_{z},$
- Group IV (very poor): $z_{i}<\bar{z}-s_{z},$

where:

*z_i_*—the value of the indicator in the *i*-th period of time,
$\bar{z}$—arithmetic mean of the index,
*s_z_*—standard deviation of the index.

4 typological groups of objects analysed were identified—very good, good, poor and very poor—due to the degree of the phenomenon on the basis of the value of the arithmetic mean and standard deviation in individual synthetic measures, confronted with the index value for a given country.

This paper additionally applies a more extended procedure of classification of countries using two methods of statistical analysis:

- Factor analysis, by means of the Principal Component Analysis—PCA;
- Cluster analysis (by means of the method of k-means, another multivariate comparative analysis was used).

In order to verify whether there is a point in carrying out a Principal Component Analysis, the Bartlett’s Test of Sphericity was used, and so was the Kaiser-Meyer-Olkin measure (KMO); a KMO value below 0.5 was considered unacceptable, while a reading of above 0.8 was ideal [102].

The selection of the number of components was determined by the following facts:

- The Kaiser criterion—further analysis must use only those components whose eigenvalue is greater than 1 [103];
- A scree plot test—a point is marked on the line graph of eigenvalues at which the eigenvalue drop stabilises (this analysis does not give unambiguous information, whereby this criterion must be treated as an auxiliary or supplementary element) [104].

The second analysis used, the cluster analysis, is a method of so-called classification without supervision. It is used to group elements into relatively homogenous sets. The basis of the grouping in most of the algorithms is similarity between elements, expressed by a similarity function (measure) [102,105]. The distance (between two objects x_i_ and x_k_) may be calculated in a number of ways. This article relies on calculating the Euclidean distance between individual objects on the basis of this formula:

(7) $$d(x_{i},x_{k})=d_{ik}=\sqrt{\sum_{j=1}^{p}(x_{ij}-x_{kj}{)}^{2}}$$

where:

*d*—Euclidean distance,
*x_ij_*—value of object *x_i_* in relation to feature *j*
*p*—number of features.

This method serves to group observations on the basis of an analysis of variables which are poorly correlated.

**Figure 1:**
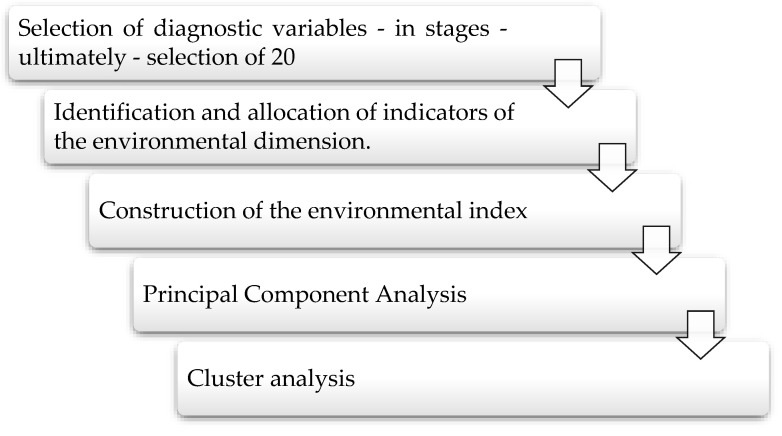
Graphic presentation of the research methodology.

Figure 1 shows a graphic presentation of the research methodology.

## 3. Results and Discussion

The EI created allows a comparison of Poland’s position (as well as of the position of other countries) in terms of its progress in the implementation of actions in the natural environment area with other Union countries. The value of EI (EI formulated according to the description in part 2) obtained by each of the countries analysed was the basis for marking their position in the ranking of EU-28 countries (Table 4).

Denmark and Sweden scored the highest EI values in the EU-15 group, and among all of the countries analysed. In turn, in the EU-13 group, the highest EI values were noted for Latvia, which came third in the overall classification of EU-28 countries in 2010, and fourth in 2020 (Table 4).

The lowest values for EI, and thus the last places in the ranking, went to Romania (which came 28th in the perspective of the years examined) and to Bulgaria, whereas in the EU-15 group, these places were taken by Luxembourg, Italy and Greece. Compared to the remaining EU-28 countries, Poland was 21st in 2010 (ahead of Greece or Luxembourg of the EU-15 group) and 23rd in 2020.

The EI had a rising trend for Poland in the period analysed. 2020 recorded a growth of 16.7% compared to 2010.

An analysis of compliance with the ordering in subsequent years was carried out for the EI using the Spearman rank correlation coefficient. The values of the coefficients point to a high compliance with the ordering in subsequent years (Table 5).

An arithmetic mean ($\bar{z}$) and standard deviation (*s_z_*) were used to assign the countries to one of four groups (part 2 of this article) depending on their EI level. Group I accommodated countries with the highest EI values, while group IV had those for which the EI value was least favourable. Table 6 presents a joint compilation of countries in separate groups for individual years.

When analysing the classification of countries under specific groups, it is difficult to point to marked regularities among the EU-13 and EU-15. All four groups have countries of the “old Union” and those newly received. However, it is noticeable that, out of the “old Union” countries, groups I and II were allocated countries which had, for a long time now, been actively involved in environmental actions or actions for rational resource management. These countries also run an active ecological policy (which, in Poland, has only been implemented since 1989); for example, Denmark, Sweden, Finland, the Netherlands or Germany do so.

We must emphasize that, even though Poland featured in group IV in the final years of the analysis, it took up projects throughout the period analysed to protect the natural environment, which could translate to the EI’s rising trend observed over the research period.

After a base classification of the EU countries, a more extensive procedure of their grouping by means of the Principal Component Analysis and a cluster analysis (by means of k-means clustering) was carried out. This was done for further in-depth analysis and to verify this analysis to show its reliability.

The Principal Component Analysis may be carried out on variables that are strongly correlated with one another and which then are replaced with one factor. The weights (loads) are bound with the basic variable by the strength of association with the base variable. In the case analysed, the assumption was to carry out the Principal Component Analysis in blocks of variables for the EI. Individual sets of variables are values of a given coefficient in individual years (Table 7).

The analysis of the data included in Table 7 shows that a (positive) correlation between its values in individual years is very strong—the correlation coefficients were between 0.941 and 0.996. Therefore, we may assume that these variables may be used for further analysis.

Bartlett’s test of sphericity and the KMO index were used to verify whether there is a point in conducting a component analysis for a given set of variables (values of individual indicators in 2010–2020).

The results obtained prove that a factor analysis is recommended (Table 8). Measures of the adequacy of the sample selection were above the 0.8 threshold.

**Figure 2:**
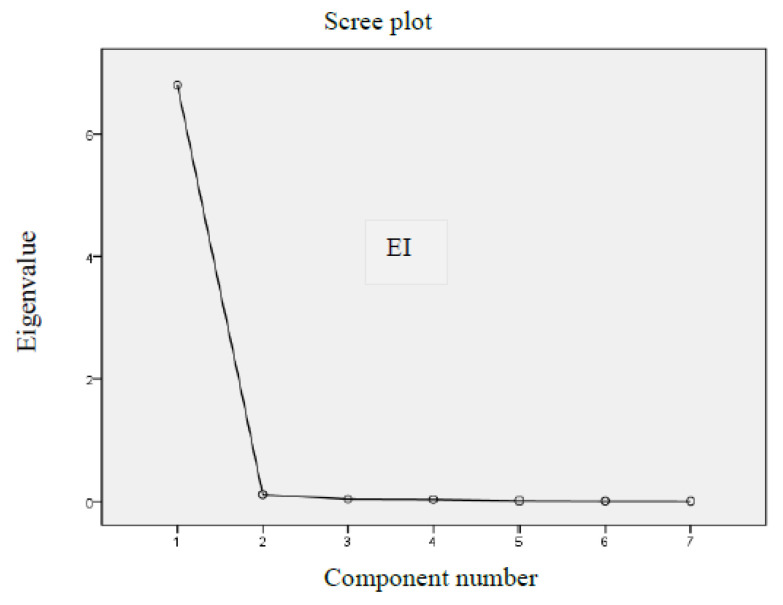
Scree plot for the EI. Source: authors’ own compilation.

Another step in the analysis was to determine the factors (on the basis of a scree plot—Figure 2).

The eigenvalues of the factors and the percentage of the sum of the variance explained were also specified (Table 9).

The following conclusions may be drawn from this analysis:

The scree plot for the EI suggests one factor. The Kaiser criterion also points out that only the first factor (eigenvalue greater than 1) must be used for further analysis. This factor carries 97.2% of the information included in the input variables.

Then, the values of the components selected for the analysis were calculated for individual EU countries (Table 10). As a result of this work, the group of values of the coefficient (index) in 2010–2020 was reduced to one factor, and thus the procedure now produces a variable that explains the indicator in the period of eleven years with minimal information loss.

The value of the environmental area factor was one of the lowest for Poland in comparison with the remaining EU-28 countries, which directly translated to the position taken by Poland, both in the general ranking (23rd place among the EU-28) and in the EU-13 group (9th place). Among the EU-28 countries, the value of the environmental area factor was the highest for Denmark and the lowest for Romania. The highest value here among the EU-13 countries was noted for Latvia (thirds place in the EU-28), whereas Greece scored the lowest for this component in the EU-15 group (24th place in the EU-28).

When comparing the ordering of the countries in terms of their EI value in 2020 and in terms of the value of the environmental area factor, we may also note a certain correlation. Those countries which were classified in the ranking of the EU-28 countries, both the highest and the lowest in the case of the environmental index, took the leading or weakest positions in the ranking of the value of the environmental area component, respectively.

Ultimately, in order to group the 28 EU countries, a cluster analysis by k-means clustering was employed. It was assumed that the countries would be classified into two clusters, which shed a new light on the EU states belonging to specific groups. The relevant data are presented in Table 11. The third column presents the Euclidean distance from the cluster center.

The final centers of both clusters are presented in Table 12.

This division into two clusters seems most correct in this analysis because the countries studied in this paper are also examined in the context of belonging to two groups, that is, the EU-13 and the EU-15. By doing so, we may look at this belonging from a different perspective. This analysis showed that two countries that form part of the EU-13 found themselves in the group with the best countries of the “old Union,” creating the first cluster with them. They were Latvia and Estonia. At the same time, 7 countries of the “old Union” were in the second cluster along with 11 countries that make up the EU-13, including Poland. It is worth pointing out that the degree of progress in Poland’s actions for the natural environment is relatively higher than in some countries of the “old Union” which make up the second cluster with it. Therefore, we may conclude that a long-term presence in the structures of the European Union does not guarantee good results for a given country in all of the dimensions analysed above.

For many years now, the EU has been making efforts to reduce the pressure on the natural environment, which may be seen in its policies (e.g., recently, the sustainable development policy or the policy on promoting sustainable growth) and documents, some of which oblige the EU countries to act for environmental protection (as is the case in, for example, the Europe 2020 strategy).

The EU accommodates countries of a differing level of social and economic development [106,107,108], and, as seen in the analysis carried out here, at different stages of advancement in terms of environmental actions.

The analysis shows that the EU-15 countries, in particular Denmark and Sweden, scored the highest among all the EU countries. On the other hand, the best-ranking country in the EU-13 group was Latvia, which in the EU-28 classification came third or fourth when it comes to the EI, depending on the year of the analysis. Greece produced the lowest values of the EI across the period analysed for the EU-15 countries, and it was Bulgaria and Romania in the EU-13 group. They also were last in the general classification of the EU-28.

In the overall assessment of the EU countries for the EI values, Poland was between the 21st and 23rd place. It is highly satisfactory. However, it must be reserved that, despite a drop in the ranking throughout the period analysed, the EI for Poland showed a rising trend (a rise by 16.7% in 2020 compared to 2020). This came down to investment projects in environmental protection, mainly in the realm of water and sewage management and the protection of ambient air. Such an increase was also noted for other EU countries.

Such a grouping (four categories identified) of countries in terms of their advancement in the environmental area demonstrated that Poland takes a place in the last, that is, IV group, in the period analysed among the EU-28 countries, alongside Croatia, Romania, Bulgaria, Slovakia, Greece, the Czech Republic, Luxembourg (in 2010–2011) and Italy (in 2012–2015). Each of these groups featured both EU-15 and EU-13 countries, but in groups I and II, the EU-15 countries prevailed. The factor analysis also showed that the value of the environmental area component for Poland, in comparison with the other EU-28 countries, was one of the lowest (in the EU-28 it was the highest for Denmark and the lowest for Romania). The cluster analysis revealed that Poland (along with other EU-13 countries) was given a place in cluster II, that is, a cluster with poorer results in the environmental area.

The countries which are leaders in the EI ranking, that is, Denmark and Sweden, run a pro-environment and eco-innovative economy. For many years now, they have been applying technological solutions that aim to counteract the degradation of the natural environment. These actions are based on the rational use of available resources. They also take into account tax solutions that support the protection of the natural environment [20]. The countries observe a trend to separate economic growth from the negative pressure exerted on the natural environment [109]. Generally, it is characteristic for countries that follow the Nordic model. Other EU countries, especially those that make up the EU-15 (e.g., Germany), also use the Nordic model [110].

Among the EU-13, Latvia achieved the best results in the environmental area. This is mainly due to the effective implementation of the goals of the energy and climate policy [111]. As the data show, primary energy consumption in Latvia is one of the lowest among the EU countries, whereas the share of RES in the gross final energy consumption is high. The high share of RES translates into Latvia’s drop in energy reliance (in 2020, this reliance was 45%, compared to, for example, Greece, where it was around 82%). We also observe a significant increase in the recycling of municipal waste in Latvia, from 9.4% in 2010 to 39.6% in 2020. Greenhouse gas emissions per capita also look good for Latvia—it is lower than the EU-28 average: in 2020 it was 5.6 for Latvia and 8.1 for the EU-28.

The research results show that most EU countries stay at an average level of taking actions in the environmental area. Their engagement in environmental protection actions must be considered insufficient. We may seek the underlying basis for this in many factors. They include a prime place taken by the continuation of the style of running business practices and the inefficiency of some of the measures (including legal ones) used, such as not offering many tax incentives for pro-environmental unertakings, slowly introducing changes in environmental policy (including elements that relate to energy and climate change), or subsidising sectors that burden the natural environment. Given the above, it is necessary to encourage the EU countries to have a comprehensive approach to the introduced changes that are to aid in environmental protection. The solutions introduced should be based on research that specifies the impact of major existing trends, that is, digitisation, automation and globalization, on environmental results. We must also note the reliable assessment of how individual countries apply environmental policy instruments. An important aspect also involves striving to associate eco-innovations and activities to protect the environment with various business models. This fits the terms of greening the economy and supports the achievement of CE goals and the goals of environmentally sustainable development. A diagnosis of environmental policies and of the tools applied points out what importance must be given to environmental priorities in pursuing green transformation and in achieving climate neutrality in individual EU countries. As seen in the research results, it will be one of the major challenges for most countries in the coming years. In this context, we must also note the economic complexity specific to individual EU countries. This is associated with adopting certain solutions in economic policy [112,113], which is to translate into solutions to be incorporated into environmental policies.

Bulgaria and Romania are the countries with the worst results in the environmental area. When it comes to Bulgaria, it is quite important that more than 45% of the electricity is generated from coal, whereas RES investment is rather modest. This situation is expected to change as a results of the ambitious goals of the energy strategy. They assume, for example, the sharing of the RES sector in up to 27% of the gross final energy consumption and the development of the energy storage systems market. Energy poverty in Bulgaria remains a major challenge. Compared to Bulgaria, Romania has less electricity generated from coal (approx. 24%), however, energy from RES amounts to 40% there. Nevertheless, the problem lies in the not-fully-used RES potential, mainly due to the minimal investment in this sector and due to the dispersed location of resources or environmental limitations [114,115].

The results of the analysis showed that Poland demonstrated a relatively poor degree of advancement against all the EU countries analysed in the environmental area. Solid fuels (hard coal, lignite coal and peat) are basic energy carriers in the Polish economy, in contrast with France, where nuclear energy dominates. Given the structure of energy carriers, the rate of greenhouse gas emissions per capita in Poland was high throughout the entire period analysed compared mainly with all countries in the EU-13 group. Nevertheless, highly developed Member States from the EU-15 group showed the highest values of this indicator. A declining trend of greenhouse gas emissions per capita was noted in all the states analysed in the research period, which was due to having to reduce emissions for reasons resulting from the EU’s energy policy. It must be reserved that the dynamics of the drop in greenhouse gas emissions per capita in Poland was not high. It was nevertheless possible thanks to, for example, restructuring the Polish energy sector and upgrading production lines in “carbon-intensive” sectors.

Regulations adopted by the European Union on the one hand impose limits of greenhouse gas emissions, and on the other, set up a threshold of using renewable energy sources (RES) in the final energy balance (e.g., 15% for Poland until 2020). As a consequence of the regulations adopted in the years analysed, we may see, for example, an increase in the share of renewable energy in the gross final energy consumption of most of the EU-28 countries. Poland recorded an upward movement of this indicator, which is due to its great RES potential, significant amounts of renewable resources (biomass or thermal waters) and favourable climate and geographical determinants (e.g., in the context of using the potential of wind turbines). The share of RES in Poland’s energy balance was relatively low during the research period. High costs are the main reason why an RES market is still doing poorly in Poland. Nevertheless, the country does show a positive trend towards an RES orientation.

Poland’s high energy dependency is another indicator that contributes to Poland’s poor position in the environmental area dimension against the EU-13 and the EU-15 groups. In the period analysed, Poland got significantly more dependent on other stakeholders in terms of energy. Its greater energy dependence results, for example, from a larger share of crude oil in the energy balance, which is almost entirely imported, and from a greater demand for energy. It needs to be emphasized that the EU’s economy is still an energy-consuming system.

All countries analysed in this research period faced the increasing consumption of materials and electricity and increasing amounts of waste generated (the generation of hazardous waste was particularly concerning). Municipal waste is also a serious problem. One of the tasks that allows for the reduction of municipal waste is the improvement of the recycling system. Developing green consumption models in households also plays an important role and leads to a reduction in the amount of waste and to its segregation. Waste recovery and recycling is a basic form of reducing environmental pressure in the area of waste.

The percentage of recycled municipal waste in Poland was modest compared, in particular, with the EU-15 countries. Nevertheless, the noticeable rising trend of this phenomenon in the years analysed is a positive trend. Despite the growth of the percentage of recycled municipal waste, Poland was still a country with one of the lowest scores of this indicator among all the countries analysed in 2020.

This was mainly due to a lack of adequate infrastructure and a lack of a habit to segregate waste in Poland. The latter has an underlying economic explanation. There are no effective economic tools, and the green awareness of the Polish society is low. The danger associated with poor recycling-related performance (not only the recycling of municipal waste, but also industrial waste) is waste build-up, which is a threat to individual populations or biocenoses. Leachates that flow into waters and soils are another problem, and so is the secondary emission of volatile substances, which may result in harmful physical and chemical changes in aquatic and land habitats. Another problem is waste resulting from the use of natural resources by the extractive industry, which causes essential changes in the landscape.

The basic reason for a relatively low value of the EI for Poland against the EU-13 and the EU-15 countries was the largely unsatisfactory values of individual indicators that create the EI in the period analysed. However, the data analysis allows for the conclusion that Poland does show noticeable catching-up when it comes to environmental protection and rational resource management in comparison to the highly-developed countries of the “old Union” and to those countries of the EU-13 group which scored better for the EI than Poland.

## 4. Conclusions

The article assesses the degree of advancement of the EU countries in the environmental area, with a particular emphasis on Poland against the EU countries.

The basis for the assessment was the EI, built on selected indicators. It is believed that the synthetic measure better reflects the differentiation of the level of advancement of the EU countries in the environmental area than the observation of individual indicators. Its most valuable feature is an overall look at the problem analysed (the work’s practical aspect). It allows for easy comparisons of the level of advancement in the environmental area between individual countries or individual groups of countries. Apart from the creation of the EI, this paper is also of value because it takes into account two analyses, that is, the factor analysis and the cluster analysis. They apply to the EU-28 countries, though broken down into two groups, that is, the EU-13 and the EU-15. With these as a baseline, one country, i.e., Poland, was analysed, which is also this work’s essential merit.

This discussion and research carried out lead to a few general conclusions.

First of all, the subject matter accommodated in the environment area is fundamental for the functioning of the economy and life quality (socio-economic development). At the moment, a focus on a low-emission economy (for example, replacing or supplementing non-renewable energy with green energy), a resource-efficient economy (with an increased demand for natural resources that grows quickly due to the increased size of the population) and an economy with a circular focus (following CE principles) is necessary to maintain an adequate (that is, sustainable) level of socio-economic development.

Secondly, numerous EU strategies and initiatives aim to achieve sustainable economic development in a long-term perspective, which requires constant and consistent efforts to reduce the pressure on the natural environment.

Thirdly, after taking the measurements and after constructing tools that relate to SD or CE, or selected areas, including the environmental area, having an awareness of the obstacles that one may come across is important. For the environmental area, these include, e.g., limited information about differentiated natural resources, insufficient political stability required to promote the actions minimising the negative impact of entities on the natural environment (including, e.g., those relating to green processes, technologies and the green industry), and unsatisfactory general institutional support for pure energy, technology and a green economy.

Fourthly, generally positive trends are observed in shaping environmental indicators throughout 2010–2020 in the EU-28; this also translated to the observable increase in the EI value, which was based on selected environmental indicators. The EI value was higher in the EU-15 countries for many years that included intensive actions for the protection of the environment, such as Denmark and Sweden. In turn, Greece, Italy and Luxembourg scored the lowest against other countries. The lowest results in the rankings for the value of the EI throughout 2010–2020 went to Romania and Bulgaria. In general, we may conclude that the countries of the EU-13 group are less advanced in their actions for the protection of the natural environment. The cluster analysis showed that, out of the EU-13, only Latvia and Malta were accommodated under cluster 1 (countries that are advanced in terms of their actions in the environmental area). Therefore, the research showed the existing disproportions among the European countries in terms of their results in the environmental area. While most EU-15 countries obtained high results and sat at high positions in the EI ranking, new EU members still need to improve their environmental indicators.

Fifth of all, Poland scored low for the EI in the period analysed compared to the EU-28 countries and thus sat at an unsatisfactory position in the general classification—which translated to it falling under selected groups (IV) and clusters (2). This was mainly due to gross negligence in the environmental protection area before the transformation period and due to the Polish economy (especially the energy sector) relying on hard and lignite coal.

Sixth of all, today’s complicated determinants and external and internal circumstances make it difficult to assess the EI’s further development for Poland against the EU-13 and the EU-15 group and the developmental possibilities of the Polish economy. Poland surely still needs to face tasks relating to European integration and, most of all, narrowing the developmental distance that still separates Poland from the highly-developed EU countries, including in the environmental area.

A seventh point stipulated that dealing with environmental issues today is a key challenge and a vision for the coming years.

To sum up, we need to conclude that the engagement of EU countries in environmental actions is diverse and depends on the degree of economic development of the individual states. Countries that scored high in the EI ranking are strongly involved in actions for green transformation.

The inclusion of the EU countries in sustainable environmental development requires that most of them introduce numerous political, regulatory, legal and economic changes, and make major investments. It seems that a good direction to prioritize actions that serve the natural environment would involve numerous aspects. They include effectively using the EU’s structural funds for the development of new RES-associated technologies and eco-innovations; increasing the energy independence of the EU countries through the sustainable development of RES; introducing a system of dedicated subsidies and/or tax reliefs for actions that serve to promote environmental protection for companies and organizations; promoting CE principles; or raising societies’ ecological awareness.

The author is aware of the limitations of this paper, mainly the length of the research period (determined by the availability of data) and the resignation from adopting additionally qualitative indicators. It also needs to be borne in mind that measurement in the environmental area is difficult and carries a risk of error. Moreover, the final result will also derive from assumptions that must be made for the analysis.

The investigation performed in this paper may be further developed by, i.e., expanding the analysed time frame (depending on the available data), selecting other indicators, applying other statistical methods or constructing econometric models. The constructed EI may be a starting point for building other indices (e.g., a social index or an economic index), which make it possible to specify the degree of achievement of, e.g., the concept of sustainable development or a circular economy in the EU countries, but only there.

## Figures and Tables

**Table 1:** SDG indicators of the environmental area in the context of EU policy targets.

SDG	SDG Indicator	Definition. Targets. Defined by Policy Documents	Unit	Interrelation with the EU Policy—Documents.
SDG 7 -Affordable and clean energy	SDG 7_40—Share of RES in gross final energy consumption	The share of renewable energy consumption in gross final energy consumption according to the Renewable Energy Directive. Increase in the share of RES in gross final energy consumption by at least 32% by 2030	Number, energy fraction of RES (%).	Directive (EU) 2018/2001
SDG 11 Sustainable cities and communities	SDG 11_60—Recycling rate of municipal waste.	The mass fraction of recycled municipal waste divided by the total municipal waste arising. Increase in the preparation for re-using and recycling municipal waste to a minimum of 60% by weight by 2030	Number, mass fraction of recycled municipal waste.	Directive (EU) 2018/851
SDG 13 Climate action	SDG 13_10—Greenhouse gas emissions	Total national emissions of greenhouse gases (GHG), including CO_2_, CH_4_, N_2_O, F-gases (NF_3_), SF_6_ from all sectors of the GHG emission inventories. Reduce net greenhouse gas emissions by 55% by 2030 compared to 1990	Number, mass flow rate of Greenhouse Gas Emissions per capita, (kg/per year).	European Climate Law

**Table 2:** Likert scale used in the research.

Score	Definition
5—Very important	Has a direct impact and must be resolved.
4—Important	Has an impact and is relevant.
3—Moderately important	May have an impact and is somewhat relevant.
2—Insignificant	Has little impact and insignificantly relevant.
1—Not important	Has no impact or relevance.

**Table 3:** Diagnostic variables used in building the environmental index.

Variable Identification	Description of the Diagnostic Variable
X1	Generation of municipal waste per capita
X2	Recycling rate of municipal waste
X3	Renewable energy share in gross final energy consumption
X4	Energy dependence
X5	Greenhouse gas emissions per capita

Source: authors’ own compilation.

**Table 4:** The value of EI for EU-28 along with the EU-28 ranking according to EI in 2010–2020.

Country	2010		2011		2012		2013		2014		2015	
	Index	Ranking	Index	Ranking	Index	Ranking	Index	Ranking	Index	Ranking	Index	Ranking
Austria	0.429	4	0.424	4	0.442	3	0.444	3	0.442	3	0.453	3
Belgium	0.293	19	0.287	18	0.291	20	0.302	20	0.290	20	0.308	19
Bulgaria	0.203	27	0.203	27	0.213	27	0.219	27	0.218	27	0.218	27
Croatia	0.256	22	0.262	22	0.273	21	0.277	23	0.282	21	0.285	21
Cyprus	0.307	17	0.300	17	0.300	18	0.303	19	0.302	18	0.307	20
Czechia	0.230	24	0.229	24	0.241	25	0.259	24	0.240	25	0.251	25
Denmark	0.568	1	0.582	1	0.561	2	0.593	1	0.595	1	0.597	1
Estonia	0.391	10	0.371	11	0.331	15	0.367	13	0.334	15	0.340	15
Finland	0.413	7	0.413	7	0.411	6	0.412	6	0.409	6	0.393	9
France	0.353	14	0.354	13	0.363	12	0.378	12	0.366	12	0.369	12
Germany	0.419	6	0.416	5	0.419	5	0.441	4	0.424	5	0.428	5
Greece	0.247	23	0.253	23	0.250	24	0.276	25	0.260	24	0.269	24
Hungary	0.299	18	0.287	19	0.298	19	0.313	17	0.299	19	0.310	18
Ireland	0.382	11	0.383	9	0.410	7	0.403	9	0.407	8	0.396	8
Italy	0.270	20	0.272	20	0.272	22	0.277	21	0.272	23	0.284	22
Latvia	0.429	3	0.426	3	0.435	4	0.424	5	0.429	4	0.432	4
Lithuania	0.372	13	0.351	14	0.350	13	0.344	15	0.353	13	0.359	13
Luxembourg	0.211	25	0.212	25	0.322	16	0.315	16	0.325	16	0.321	17
Malta	0.403	8	0.409	8	0.410	8	0.410	7	0.408	7	0.404	6
Netherlands	0.392	9	0.377	10	0.382	10	0.390	10	0.388	10	0.386	10
**Poland**	**0.258**	**21**	**0.268**	**21**	**0.275**	**23**	**0.278**	**24**	**0.275**	**22**	**0.280**	**23**
Portugal	0.315	16	0.311	16	0.334	14	0.348	14	0.338	14	0.342	14
Romania	0.198	28	0.199	28	0.202	28	0.209	28	0.209	28	0.210	28
Slovakia	0.208	26	0.209	26	0.220	26	0.232	26	0.227	26	0.229	26
Slovenia	0.420	5	0.416	6	0.396	9	0.403	8	0.395	9	0.403	7
Spain	0.327	15	0.313	15	0.314	17	0.311	18	0.317	17	0.321	16
Sweden	0.549	2	0.558	2	0.574	1	0.588	2	0.578	2	0.572	2
United Kingdom	0.376	12	0.366	12	0.369	11	0.381	11	0.375	11	0.373	11
**Country**	**2016**		**2017**		**2018**		**2019**		**2020**			
	**Index**	**Ranking**	**Index**	**Ranking**	**Index**	**Ranking**	**Index**	**Ranking**	**Index**	**Ranking**		
Austria	0.442	3	0.455	3	0.468	3	0.465	3	0.467	3		
Belgium	0.288	21	0.312	19	0.317	21	0.309	20	0.314	20		
Bulgaria	0.223	27	0.235	27	0.232	27	0.218	28	0.213	28		
Croatia	0.268	23	0.279	22	**0.293**	22	0.301	22	0.307	22		
Cyprus	0.291	20	0.309	20	0.319	19	0.307	21	0.310	21		
Czechia	0.255	25	0.268	25	0.249	25	0.272	25	0.277	25		
Denmark	0.636	1	0.607	1	0.550	2	0.587	1	0.592	1		
Estonia	0.337	15	0.343	15	0.354	14	0.350	14	0.353	14		
Finland	0.415	6	0.395	8	0.398	8	0.413	7	0.418	6		
France	0.370	10	0.371	10	0.384	10	0.387	10	0.392	10		
Germany	0.426	4	0.432	5	0.444	5	0.437	5	0.439	5		
Greece	0.260	24	0.272	24	0.283	24	0.280	24	0.283	24		
Hungary	0.309	16	0.317	18	0.333	15	0.336	15	0.329	17		
Ireland	0.363	11	0.348	14	0.366	13	0.359	13	0.362	13		
Italy	0.292	19	0.307	21	0.318	20	0.321	19	0.319	19		
Latvia	0.415	5	0.436	4	0.452	4	0.446	4	0.442	4		
Lithuania	0.348	14	0.361	12	0.324	17	0.332	16	0.342	15		
Luxembourg	0.302	18	0.318	17	0.320	18	0.322	18	0.321	18		
Malta	0.407	7	0.407	6	0.409	7	0.401	8	0.405	8		
Netherlands	0.383	8	0.384	9	0.398	9	0.400	9	0.398	9		
**Poland**	**0.272**	**22**	**0.275**	**23**	**0.284**	**23**	**0.295**	**23**	**0.301**	**23**		
Portugal	0.350	13	0.357	13	0.370	12	0.368	12	0.363	12		
Romania	0.220	28	0.222	28	0.224	28	0.232	27	0.229	27		
Slovakia	0.235	26	0.239	26	0.247	26	0.242	26	0.241	26		
Slovenia	0.373	9	0.405	7	0.421	6	0.419	6	0.417	7		
Spain	0.308	17	0.320	16	0.331	16	0.327	17	0.329	16		
Sweden	0.571	2	0.566	2	0.569	1	0.570	2	0.572	2		
United Kingdom	0.362	12	0.370	11	0.377	11	0.372	11	0.375	11		

Source: author’s compilation.

**Table 5:** Spearman rank correlations for the ranking of the EU-28 countries according to EI.

	2010	2011	2012	2013	2014	2015	2016	2017	2018	2019	2020
2010	1.000	0.995	0.943	0.950	0.946	0.948	0.948	0.952	0.963	0.959	0.961
2011	0.995	1.000	0.955	0.957	0.950	0.947	0.948	0.950	0.954	0.952	0.960
2012	0.943	0.955	1.000	0.991	0.979	0.970	0.962	0.967	0.972	0.969	0.971
2013	0.950	0.957	0.991	1.000	0.988	0.977	0.978	0.984	0.986	0.979	0.984
2014	0.946	0.950	0.979	0.988	1.000	0.985	0.985	0.987	0.986	0.988	0.986
2015	0.948	0.947	0.970	0.977	0.985	1.000	0.982	0.982	0.984	0.981	0.985
2016	0.948	0.948	0.962	0.978	0.985	0.982	1.000	0.984	0.981	0.985	0.982
2017	0.952	0.950	0.967	0.984	0.987	0.982	0.984	1.000	0.985	0.989	0.987
2018	0.963	0.954	0.972	0.986	0.986	0.984	0.981	0.985	1.000	0.990	0.989
2019	0.959	0.952	0.969	0.979	0.988	0.981	0.985	0.989	0.990	1.000	9.87
2020	0.961	0.960	0.971	0.984	0.986	0.985	0.982	0.987	0.989	9.87	1.000

Source: author’s compilation.

**Table 6:** EI—grouping of EU-28 countries in 2010–2020.

	Year	2010	2011	2012	2013	2014	2015	2016	2017	2018	2019	2020
Country												
Austria		II	II	I	I	I	I	I	I	I	I	I
Belgium		III	III	III	III	III	III	III	III	III	III	III
Bulgaria		IV	IV	IV	IV	IV	IV	IV	IV	IV	IV	IV
Croatia		IV	IV	IV	IV	IV	IV	IV	IV	IV	IV	IV
Cyprus		III	III	III	III	III	III	III	III	III	III	III
Czechia		IV	IV	IV	IV	IV	IV	IV	IV	IV	IV	IV
Denmark		I	I	I	I	I	I	I	I	I	I	I
Estonia		II	II	III	II	III	III	III	III	III	III	III
Finland		II	II	II	II	II	II	II	II	II	II	II
France		III	II	II	II	II	II	II	II	II	II	II
Germany		II	II	II	II	II	II	II	II	II	II	II
Greece		IV	IV	IV	IV	IV	IV	IV	IV	IV	IV	IV
Hungary		III	III	III	III	III	III	III	III	III	III	III
Ireland		II	II	II	II	II	II	II	III	II	II	II
Italy		III	III	IV	IV	IV	IV	III	IV	III	III	III
Latvia		I	I	II	II	II	II	II	II	II	II	II
Lithuania		II	III	III	III	III	III	III	III	III	III	III
Luxembourg		IV	IV	III	III	III	III	III	III	III	III	III
Malta		II	II	II	II	II	II	II	II	II	II	II
Netherlands		II	II	II	II	II	II	II	II	II	II	II
**Poland**		**IV**	**IV**	**IV**	**IV**	**IV**	**IV**	**IV**	**IV**	**IV**	**IV**	**IV**
Portugal		III	III	III	III	III	III	II	II	II	II	II
Romania		IV	IV	IV	IV	IV	IV	IV	IV	IV	IV	IV
Slovakia		IV	IV	IV	IV	IV	IV	IV	IV	IV	IV	IV
Slovenia		II	II	II	II	II	II	II	II	II	II	II
Spain		III	III	III	III	III	III	III	III	III	III	III
Sweden		I	I	I	I	I	I	I	I	I	I	I
United Kingdom		II	II	II	II	II	II	II	II	II	II	II

Source: author’s compilation.

**Table 7:** Pearson correlation between values for a given EI in 2010–2020 (Subindex of the environment area).

Subindex of the Environment Area											
	2010	2011	2012	2013	2014	2015	2016	2017	2018	2019	2020
2010	1	0.996	0.953	0.955	0.945	0.942	0.941	0.943	0.948	0.945	0.951
2011	0.996	1	0.963	0.967	0.957	0.953	0.953	0.955	0.959	0.952	0.963
2012	0.953	0.963	1	0.989	0.972	0.972	0.974	0.973	0.975	0.979	0.976
2013	0.955	0.967	0.989	1	0.983	0.979	0.978	0.982	0.984	0.979	0.983
2014	0.945	0.957	0.972	0.983	1	0.991	0.966	0.972	0.981	0.979	0.987
2015	0.942	0.953	0.972	0.979	0.991	1	0.981	0.983	0.980	0.986	0.991
2016	0.941	0.953	0.974	0.978	0.966	0.981	1	0.979	0.984	0.989	0.987
2017	0.943	0.955	0.973	0.982	0.972	0.983	0.979	1	0.975	0.981	0.983
2018	0.948	0.959	0.975	0.984	0.981	0.980	0.984	0.975	1	0.979	0.986
2019	0.945	0.952	0.979	0.979	0.979	0.986	0.989	0.981	0.979	1	0.978
2020	0.951	0.963	0.976	0.983	0.987	0.991	0.987	0.983	0.986	0.978	1

Source: author’s compilation.

**Table 8:** Kaiser-Mayer-Olkin tests and Bartlett’s tests for the EI.

**KMO Measure of Sample Selection Adequacy**		0.857
Bartlett’s test of sphericity	Approximate chi-quadrants	474.367
	df	21
	significance	0.000

Source: author’s compilation.

**Table 9:** Eigenvalues of factors and the percentage of the sum of the variance explained for the EI.

Component	Eigenvalue	% of Variance Explained	Cumulative %
1	6.803	97.180	97.180
2	0.110	1.577	98.757
3	0.038	0.541	99.298
4	0.031	0.449	99.747
5	0.010	0.142	99.889
6	0.005	0.066	99.955
7	0.003	0.045	100.000

Source: author’s compilation.

**Table 10:** Values of selected factors in the EU countries together with medians of factors of the environmental area for EU-13 and EU-28.

Country	Factor—Environmental Area
Austria	0.926
Belgium	–0.716
Bulgaria	–1.513
Croatia	−0.987
Cyprus	–0.654
Czechia	–1.233
Denmark	2.531
Estonia	–0.079
Finland	0.507
France	0.046
Germany	0.758
Greece	–1.106
Hungary	–0.602
Ireland	0.185
Italy	–0.843
Latvia	0.792
Lithuania	–0.162
Luxembourg	–0.846
Malta	0.528
Netherlands	0.270
**Poland**	**–0.994**
Portugal	–0.225
Romania	–1.501
Slovakia	–1.526
Slovenia	0.481
Spain	–0.509
Sweden	2.348
United Kingdom	0.123

Source: author’s compilation.

**Table 11:** European Union countries belonging to clusters.

Country	Group of Countries	Euclidean Distance
cluster I		
Austria	EU-15	0.95
Denmark	EU-15	1.94
Estonia	EU-13	0.93
France	EU-15	0.92
Germany	EU-15	0.67
Italy	EU-15	2.19
Latvia	EU-13	1.55
Netherlands	EU-15	1.29
Sweden	EU-15	1.90
United Kingdom	EU-15	0.57
**cluster** **II**		
Belgium	EU-15	1.04
Bulgaria	EU-13	2.19
Croatia	EU-13	1.10
Cyprus	EU-13	0.64
Czechia	EU-13	1.99
Finland	EU-15	1.08
Greece	EU-15	1.55
Hungary	EU-13	1.00
Ireland	EU-15	1.39
Lithuania	EU-13	0.81
Luxembourg	EU-15	1.88
Malta	EU-13	1.12
Poland	EU-13	1.12
Portugal	EU-15	1.27
Romania	EU-13	2.27
Slovakia	EU-13	1.52
Slovenia	EU-13	2.16
Spain	EU-15	1.09

Source: author’s compilation.

**Table 12:** Final cluster centers.

Factor	Cluster	
	I	II
Environment area	0.69	−0.46

Source: authors’ own calculation.

## Data Availability

The data presented in this study are available on request.
